# Supplementary figures and images for: The CIC-ERF co-deletion underlies fusion-independent activation of ETS family member, ETV1, to drive prostate cancer progression
Source: eLife. 2022 Nov 16;11:e77072. doi: 10.7554/eLife.77072 (PMC9668335; doi:10.7554/eLife.77072)

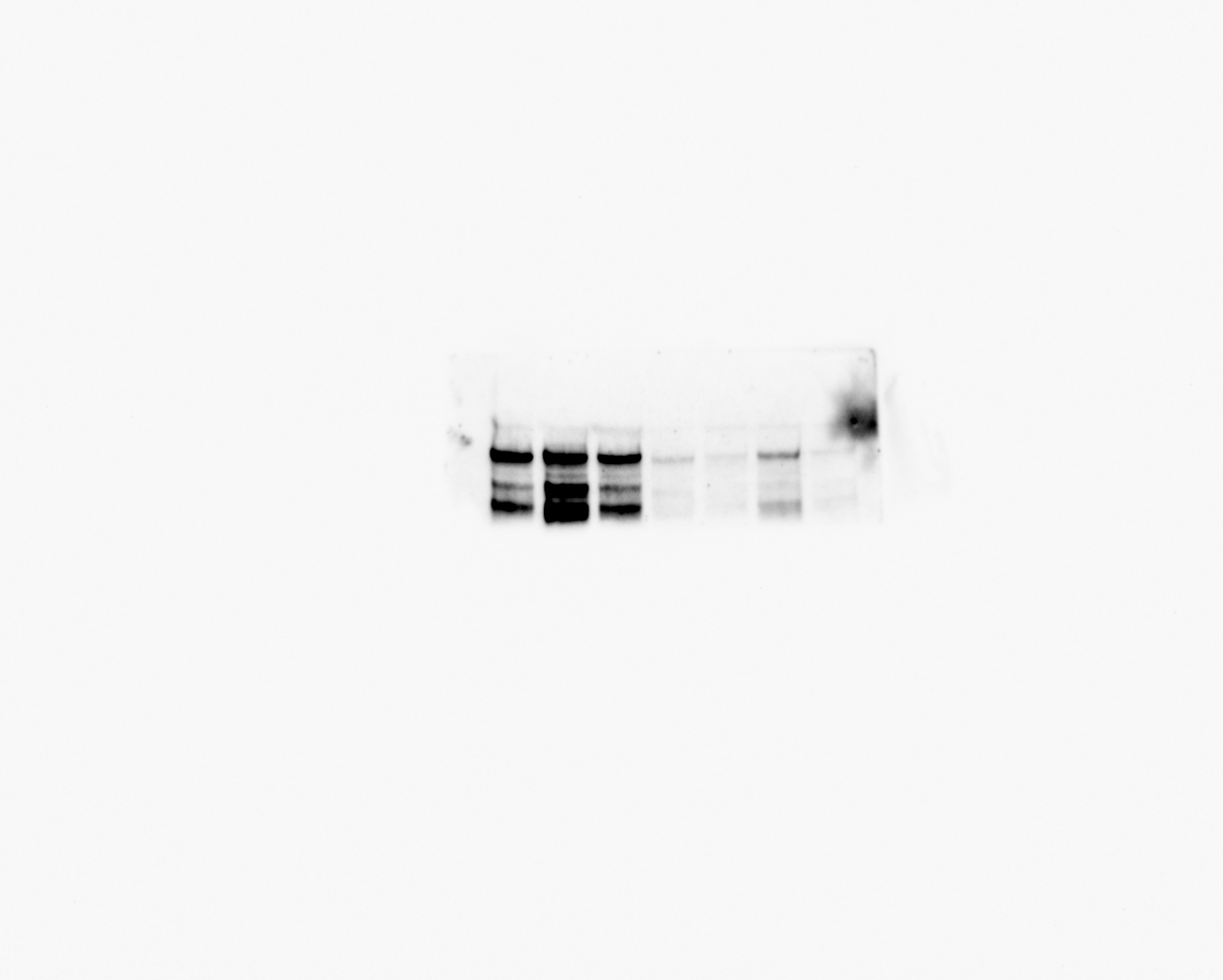

Supplement: Figure 2—figure supplement 1—source data 1. [file elife-77072-fig2-figsupp1-data1.zip › Figure 2 - figure supplement 1 - source data 1/CIC.jpg]

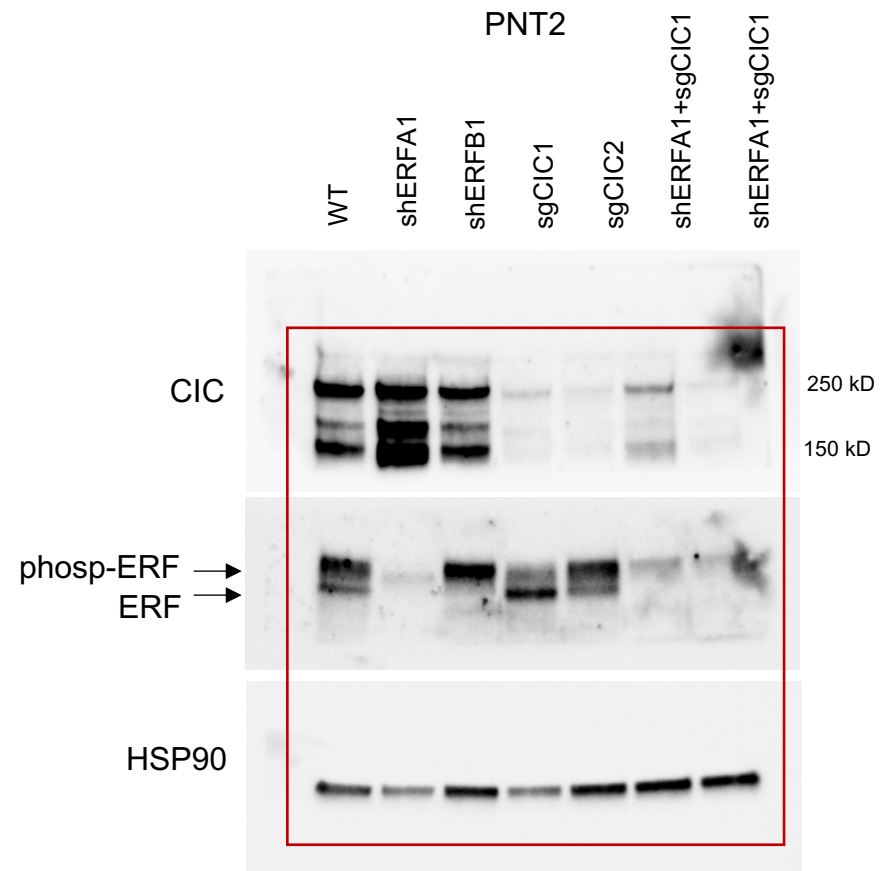

Supplement: Figure 2—figure supplement 1—source data 1. [file elife-77072-fig2-figsupp1-data1.zip › Figure 2 - figure supplement 1 - source data 1/Figure 2 - figure supplement A - Source data.pdf]

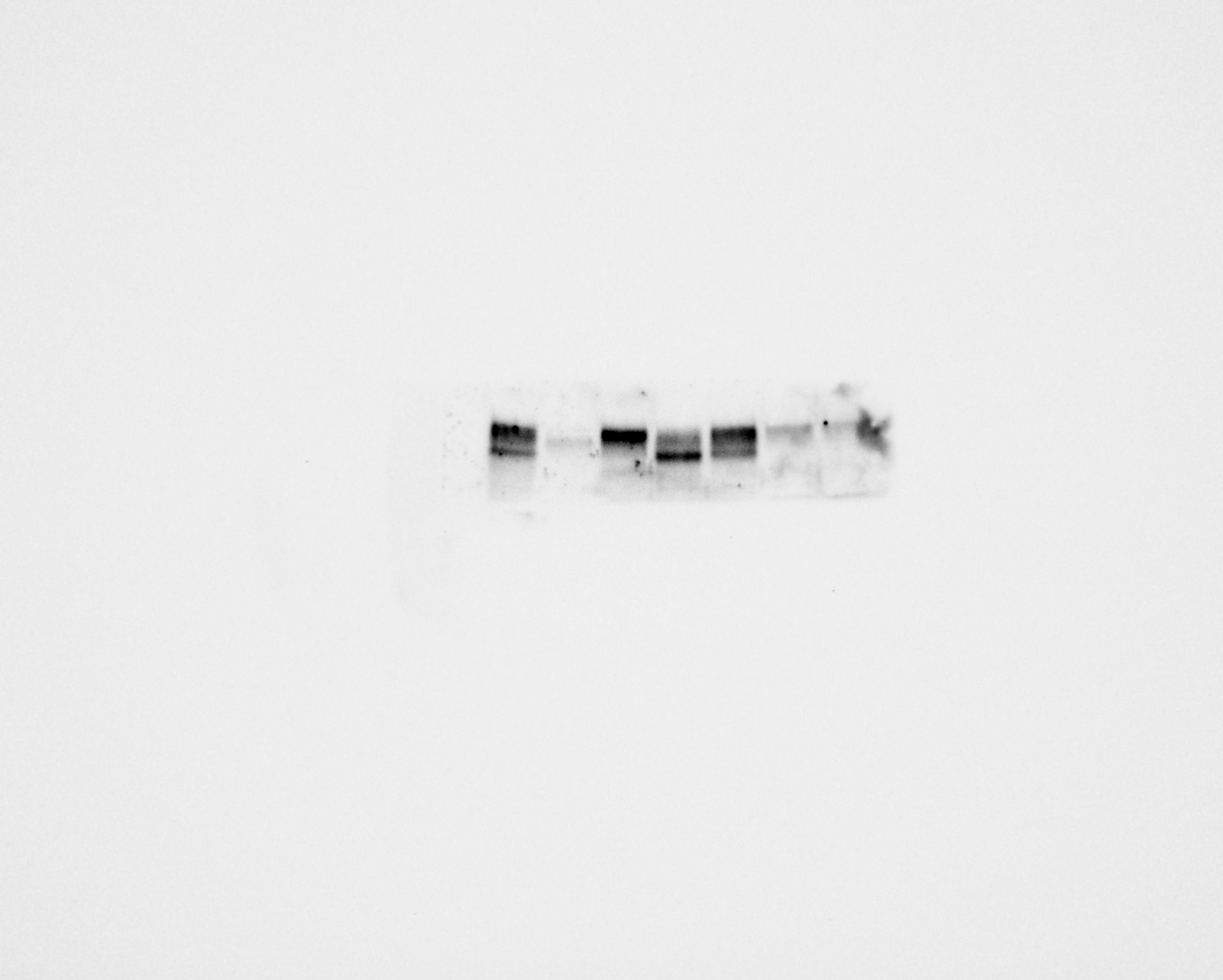

Supplement: Figure 2—figure supplement 1—source data 1. [file elife-77072-fig2-figsupp1-data1.zip › Figure 2 - figure supplement 1 - source data 1/ERF.jpg]

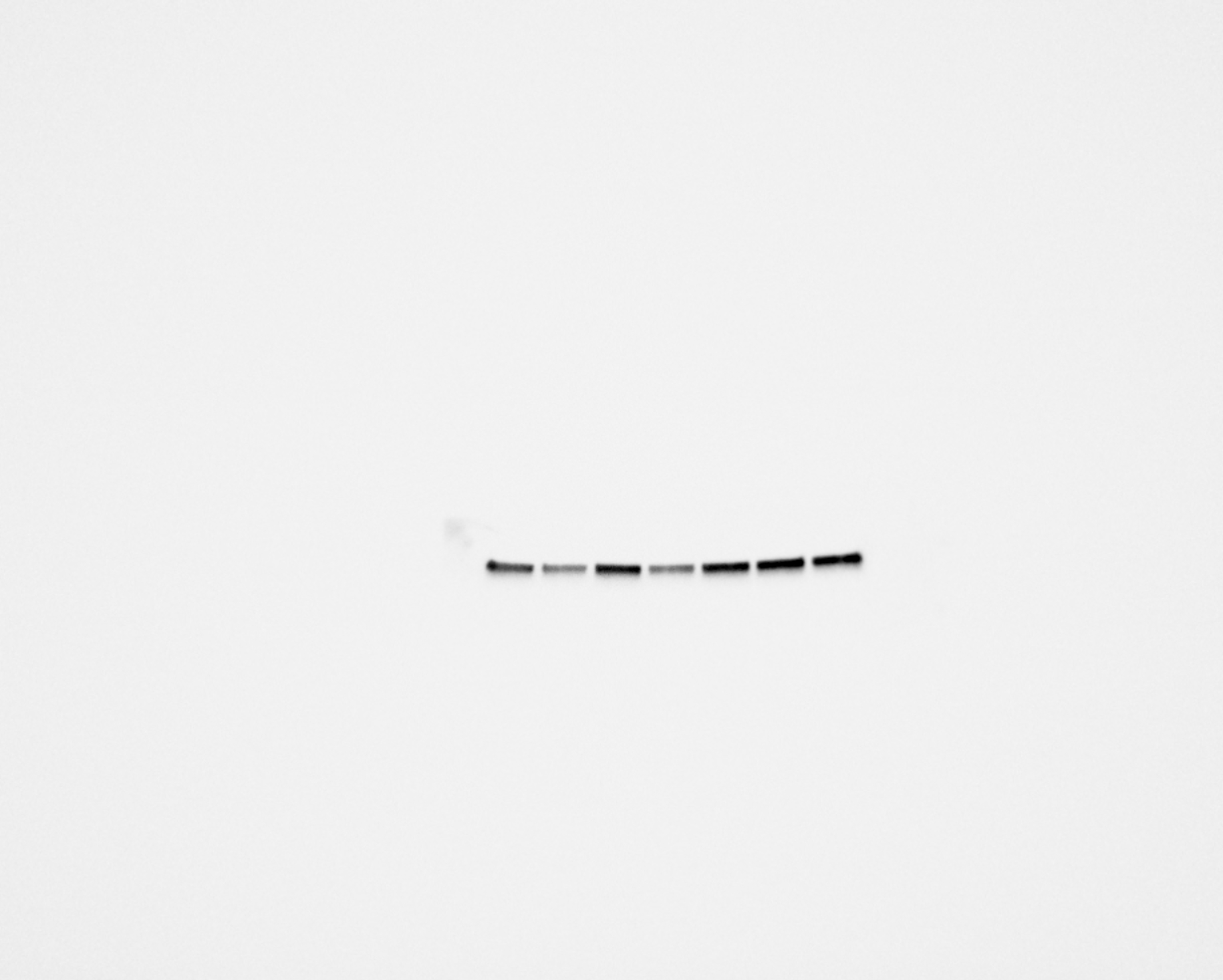

Supplement: Figure 2—figure supplement 1—source data 1. [file elife-77072-fig2-figsupp1-data1.zip › Figure 2 - figure supplement 1 - source data 1/HSP90.jpg]

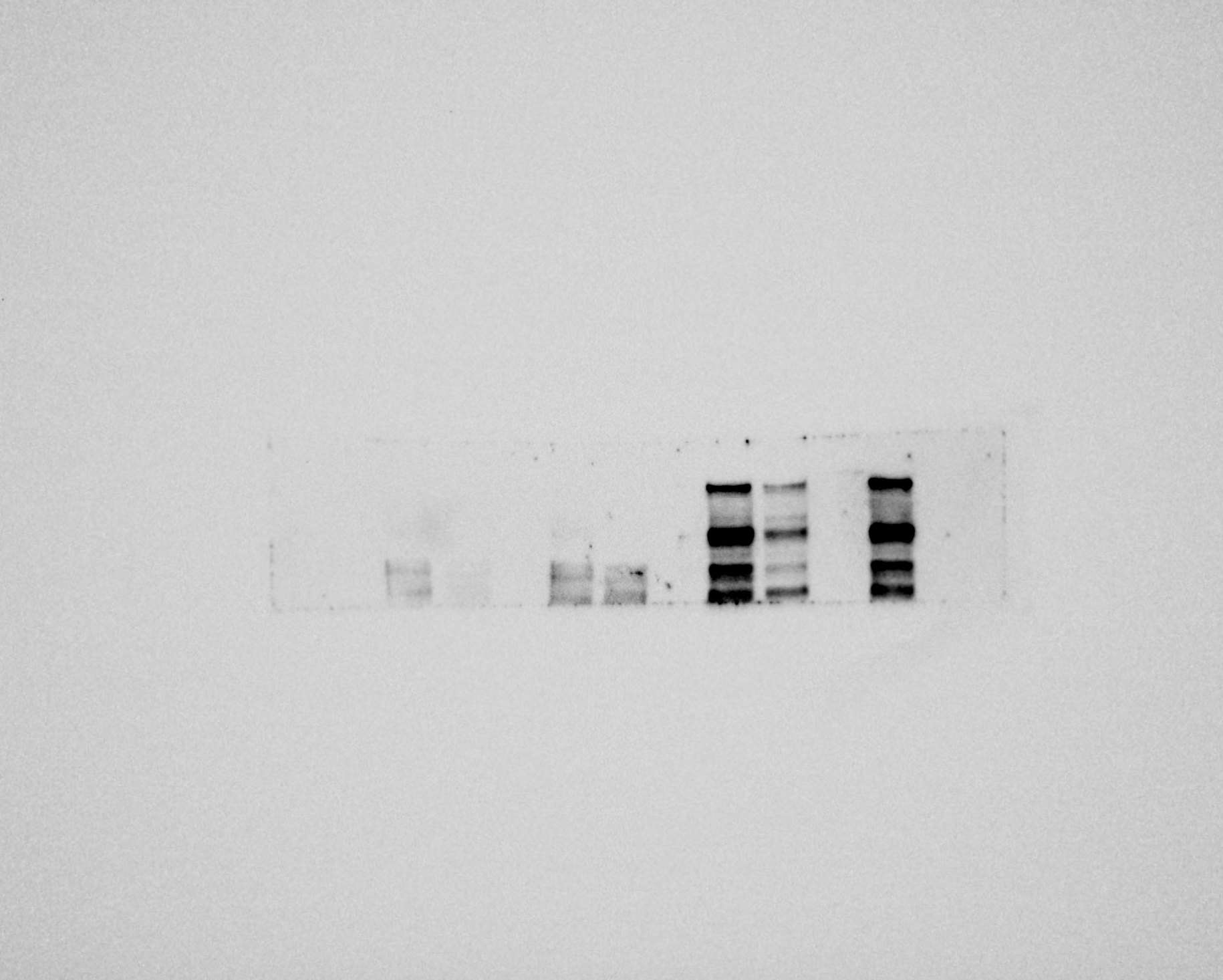

Supplement: Figure 3—figure supplement 1—source data 1. [file elife-77072-fig3-figsupp1-data1.zip › Figure 3 - figure supplement 1 - source data 1/CIC.jpg]

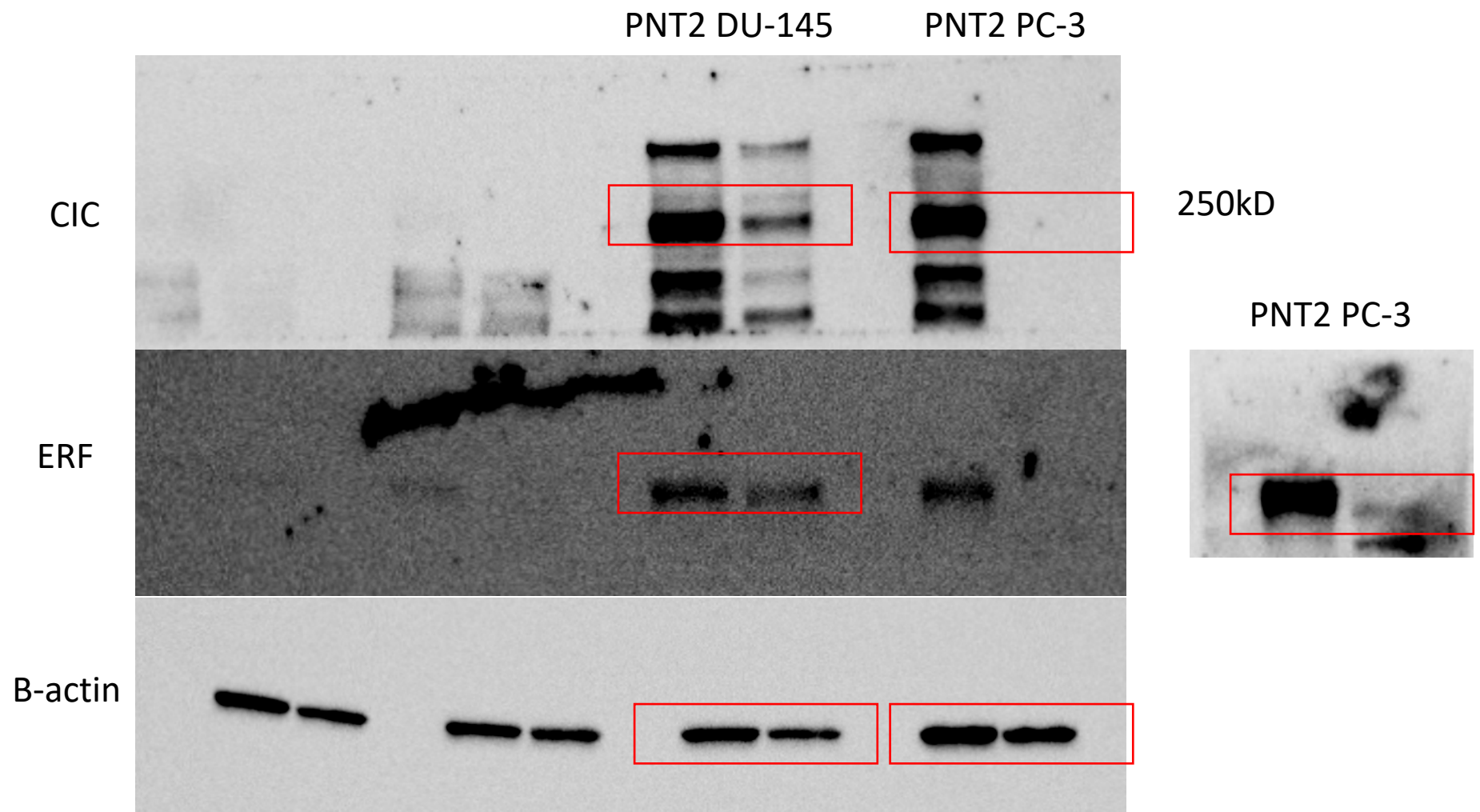

Supplement: Figure 3—figure supplement 1—source data 1. [file elife-77072-fig3-figsupp1-data1.zip › Figure 3 - figure supplement 1 - source data 1/Supplementary figure 3-figure supplement A and B-Source data.pdf]

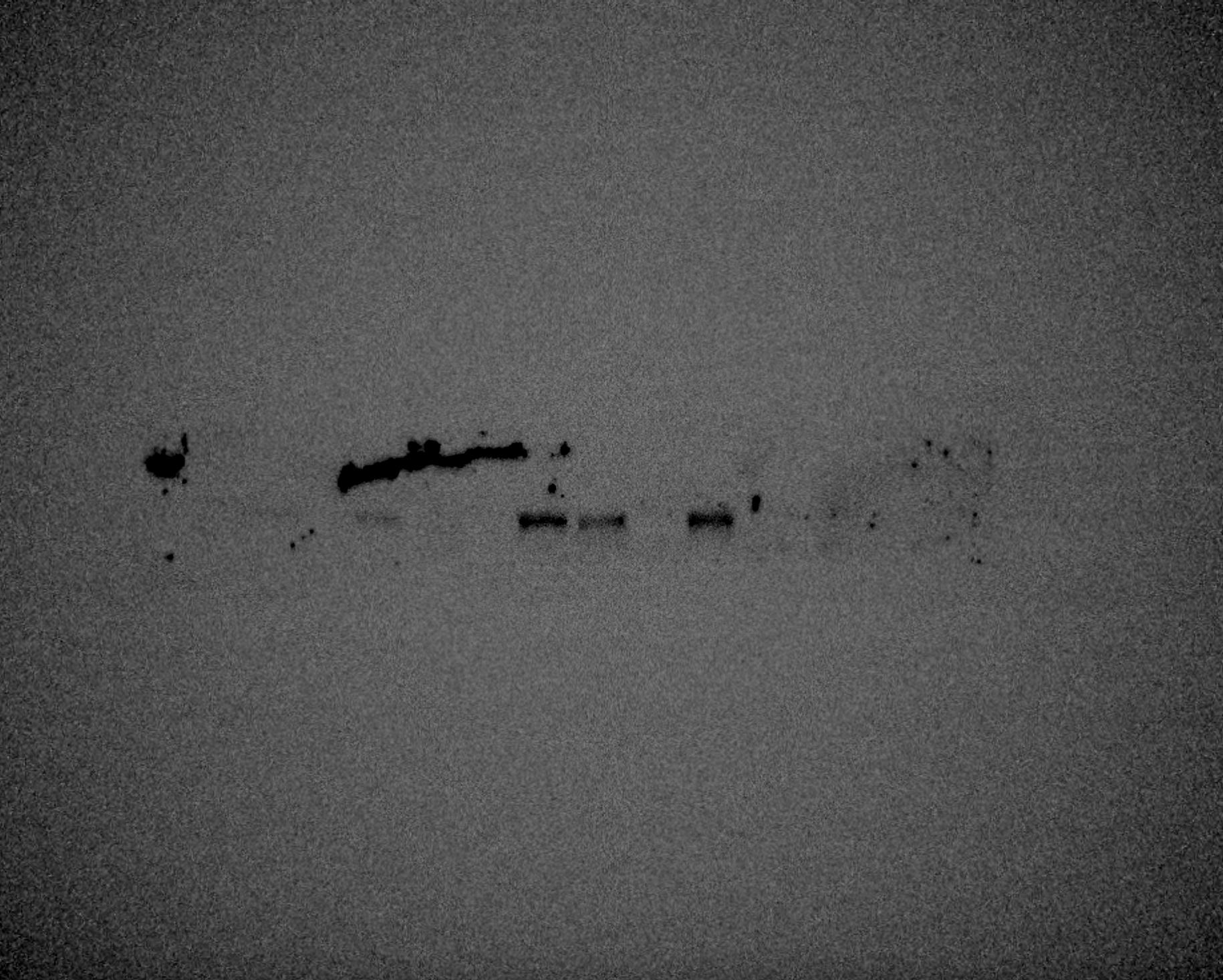

Supplement: Figure 3—figure supplement 1—source data 1. [file elife-77072-fig3-figsupp1-data1.zip › Figure 3 - figure supplement 1 - source data 1/ERF.jpg]

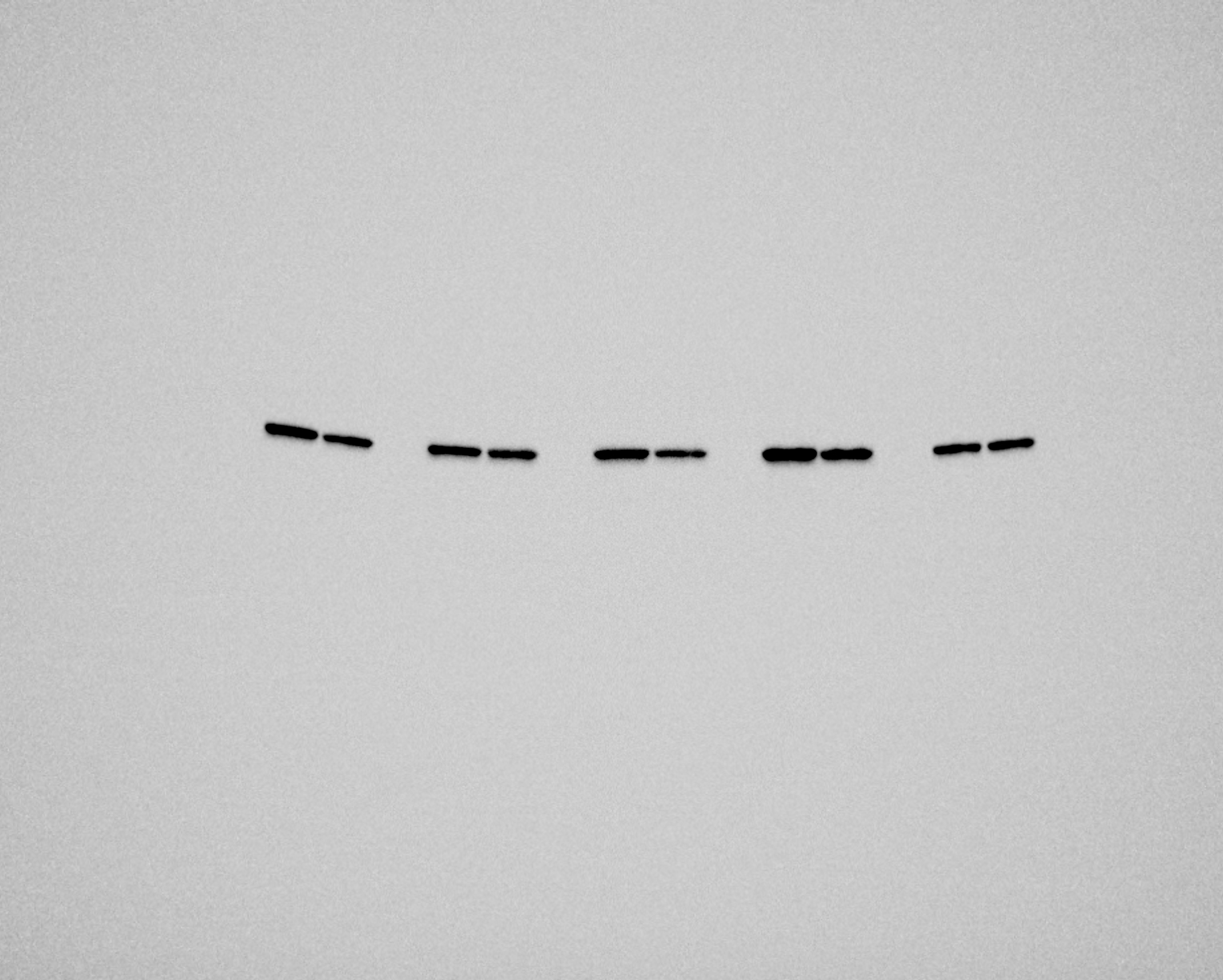

Supplement: Figure 3—figure supplement 1—source data 1. [file elife-77072-fig3-figsupp1-data1.zip › Figure 3 - figure supplement 1 - source data 1/B-actin.jpg]

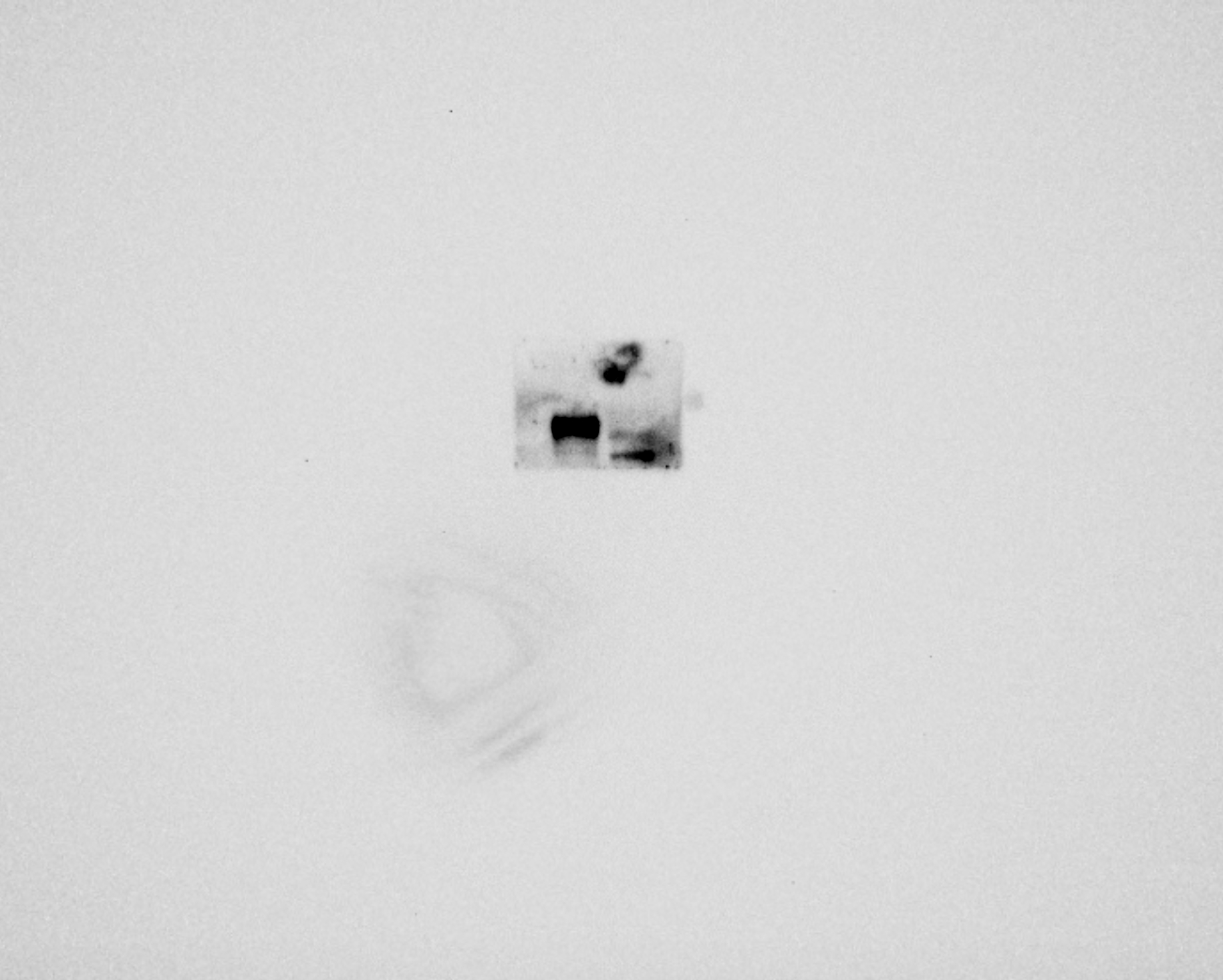

Supplement: Figure 3—figure supplement 1—source data 1. [file elife-77072-fig3-figsupp1-data1.zip › Figure 3 - figure supplement 1 - source data 1/ERF.repeat.jpg]

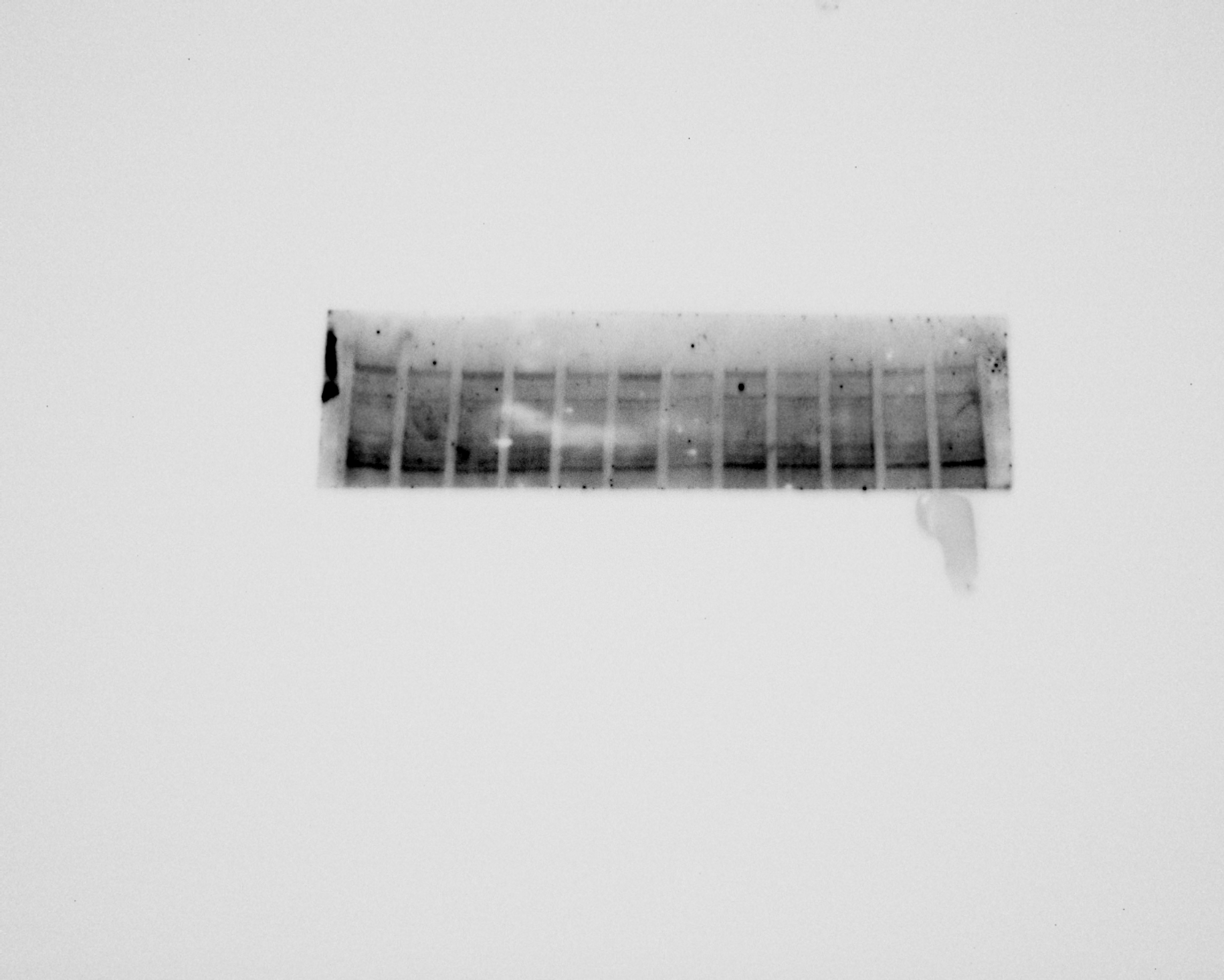

Supplement: Figure 3—figure supplement 1—source data 2. [file elife-77072-fig3-figsupp1-data2.zip › Figure 3 - figure supplement 1 - source data 2/CIC.jpg]

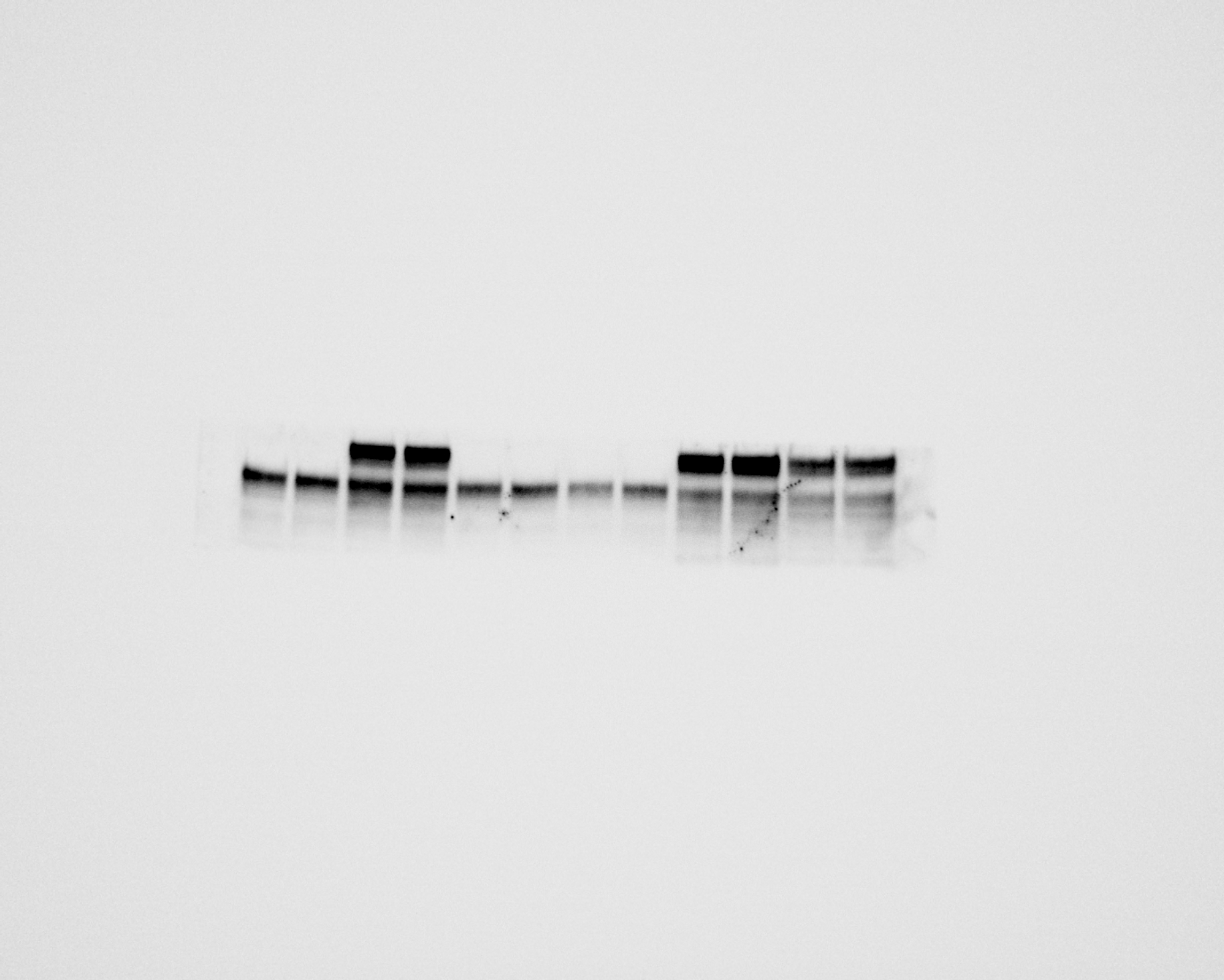

Supplement: Figure 3—figure supplement 1—source data 2. [file elife-77072-fig3-figsupp1-data2.zip › Figure 3 - figure supplement 1 - source data 2/ERF.jpg]

DU145

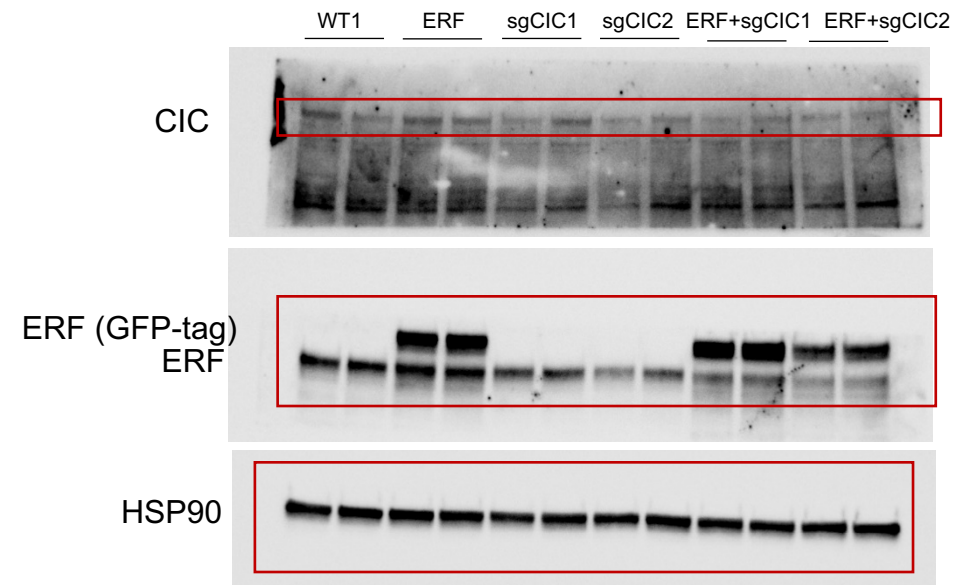

Supplement: Figure 3—figure supplement 1—source data 2. [file elife-77072-fig3-figsupp1-data2.zip › Figure 3 - figure supplement 1 - source data 2/Supplementary figure 3-figure supplement C-Source data.pdf]

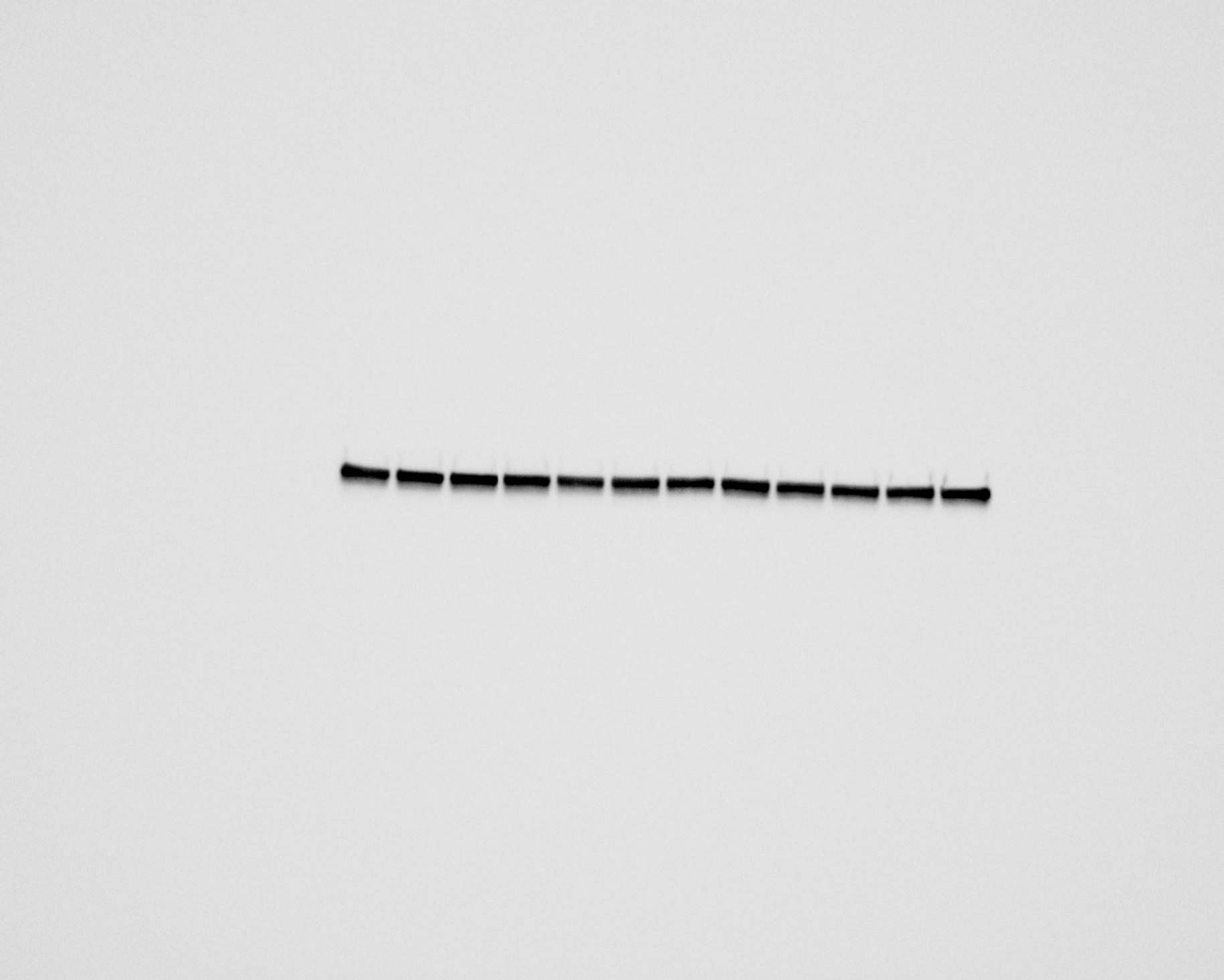

Supplement: Figure 3—figure supplement 1—source data 2. [file elife-77072-fig3-figsupp1-data2.zip › Figure 3 - figure supplement 1 - source data 2/HSP90.jpg]

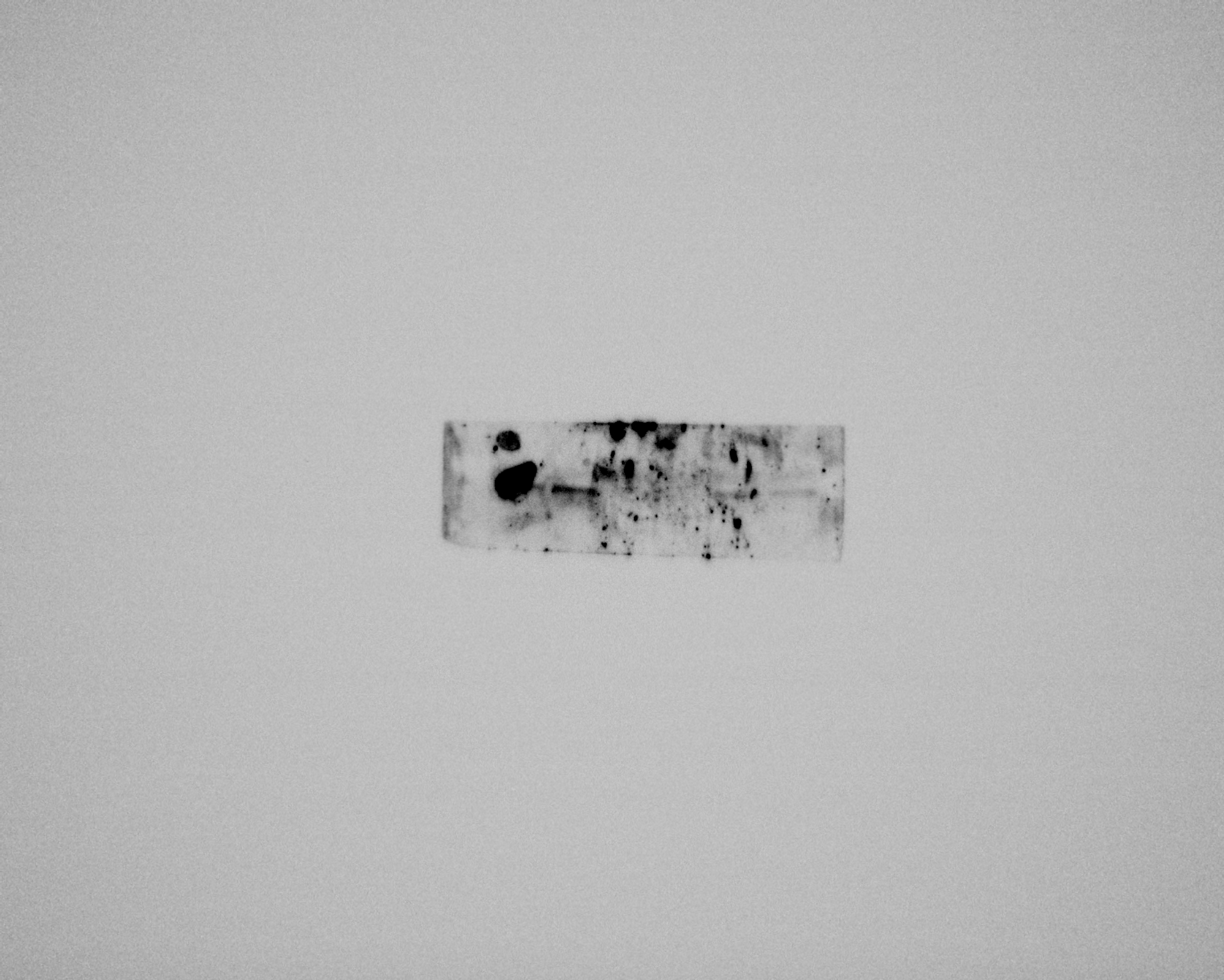

Supplement: Figure 3—figure supplement 1—source data 3. [file elife-77072-fig3-figsupp1-data3.zip › Figure 3 - figure supplement 1 - source data 3/ERF.jpg]

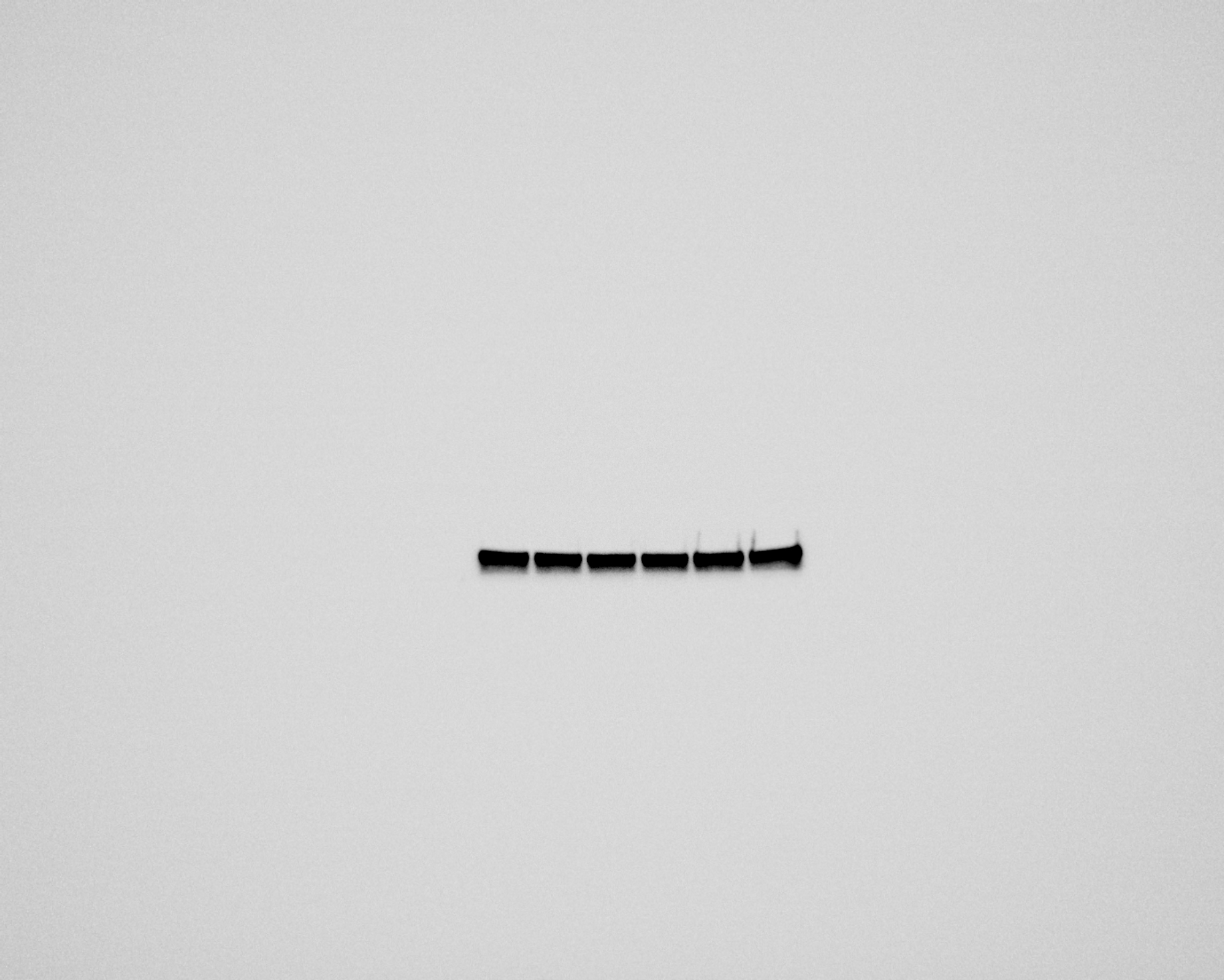

Supplement: Figure 3—figure supplement 1—source data 3. [file elife-77072-fig3-figsupp1-data3.zip › Figure 3 - figure supplement 1 - source data 3/HSP90.jpg]

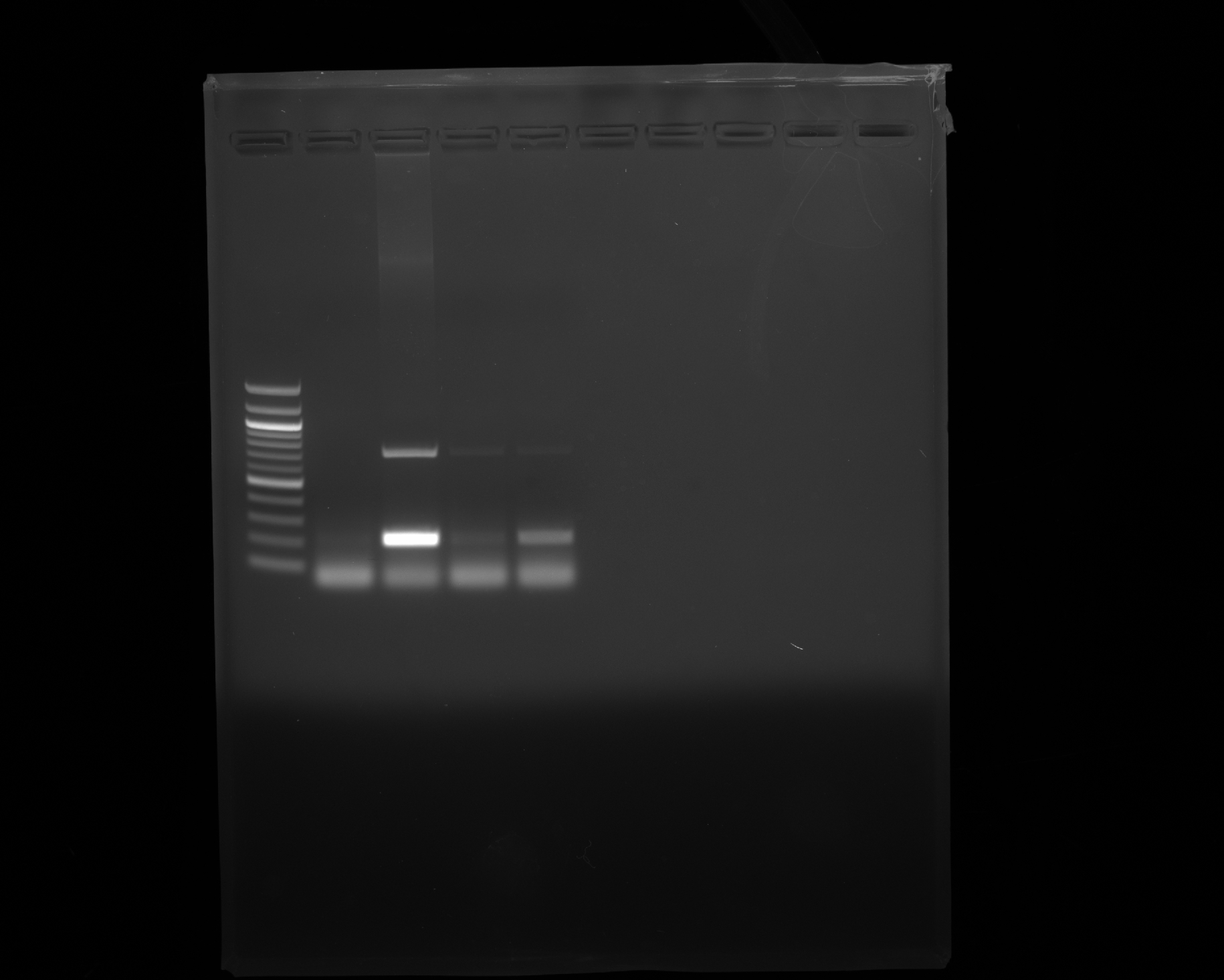

Supplement: Figure 4—source data 1. — Cropped images and description shown in Figure 4E. [file elife-77072-fig4-data1.zip › Figure 4 - source data 1/ETV1.jpg]

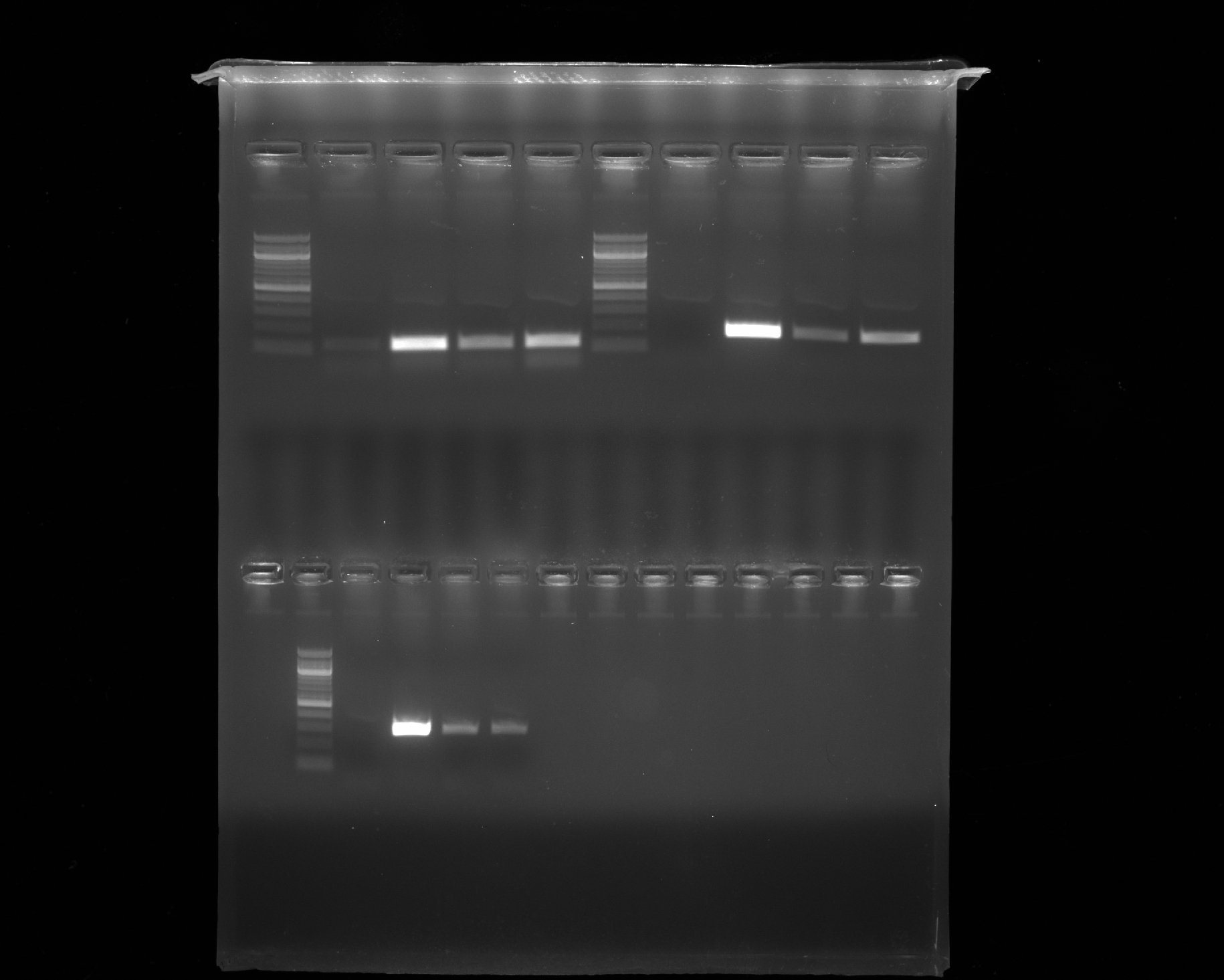

Supplement: Figure 4—source data 2. — Cropped images and description shown in Figure 4F. [file elife-77072-fig4-data2.zip › Figure 4 - source data 2/ETV1.tif]

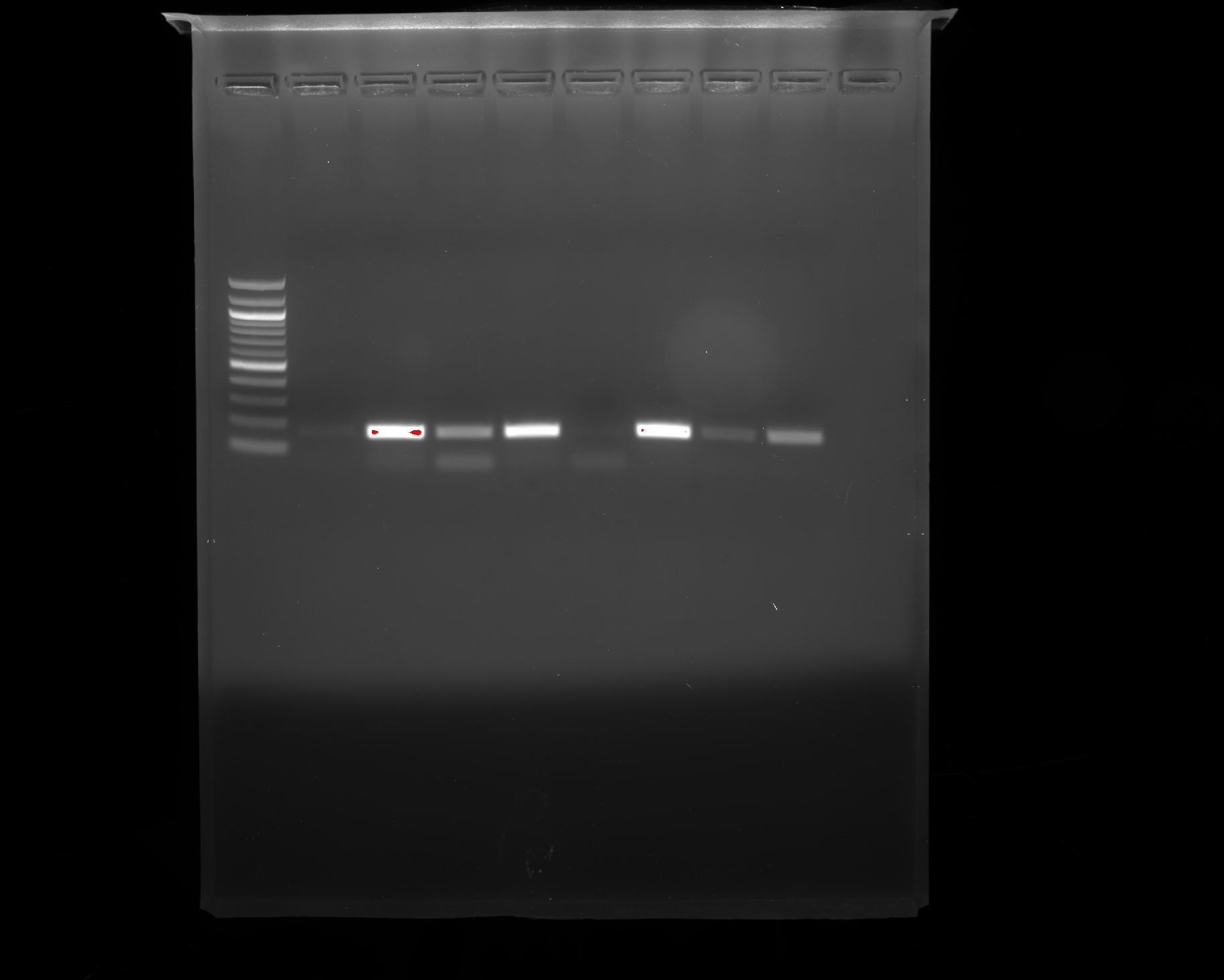

Supplement: Figure 4—source data 3. — Cropped images and description shown in Figure 4G. [file elife-77072-fig4-data3.zip › Figure 4 - source data 3/ETV1.jpg]

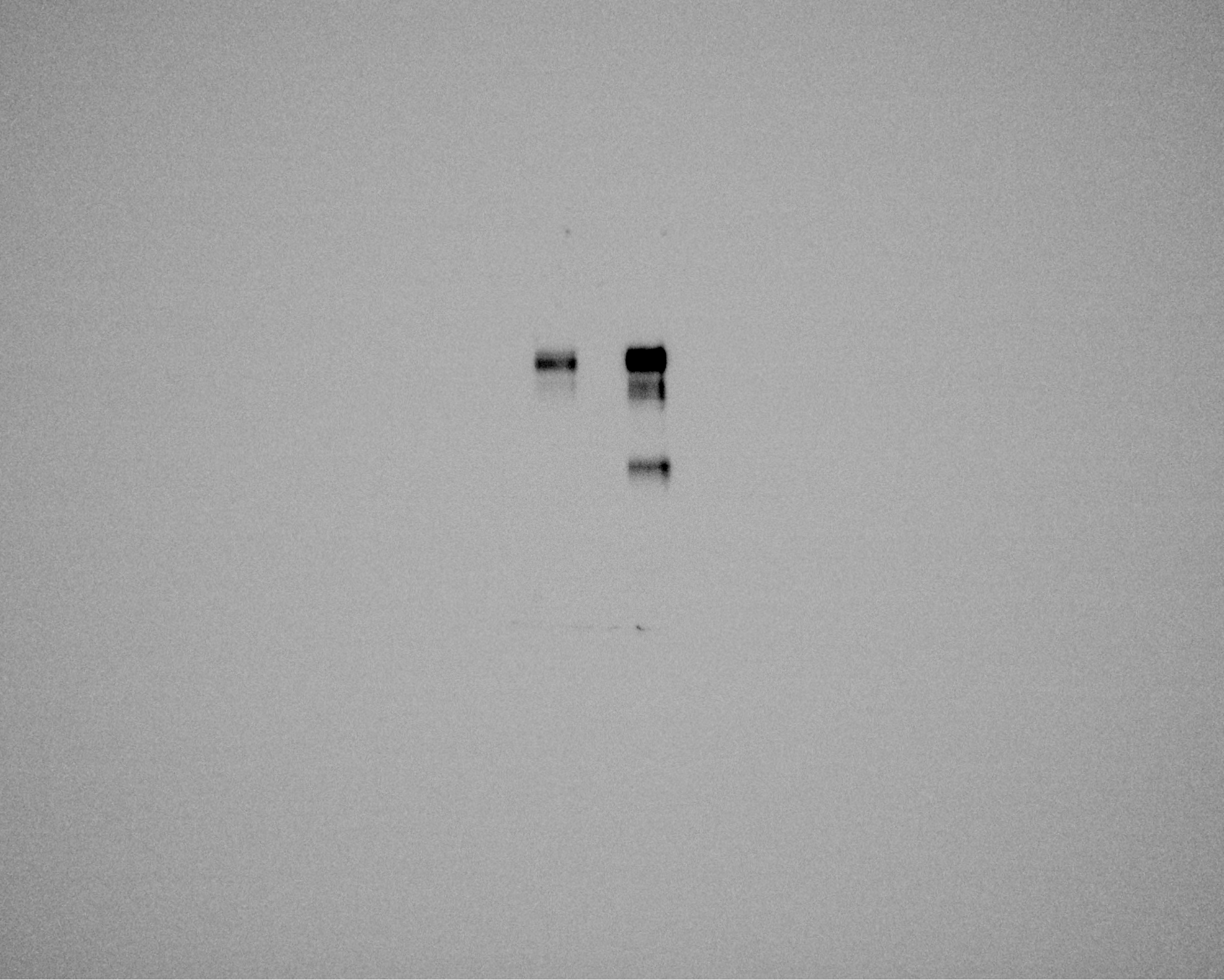

Supplement: Figure 4—figure supplement 1—source data 1. [file elife-77072-fig4-figsupp1-data1.zip › F1S1SD1a.jpg]

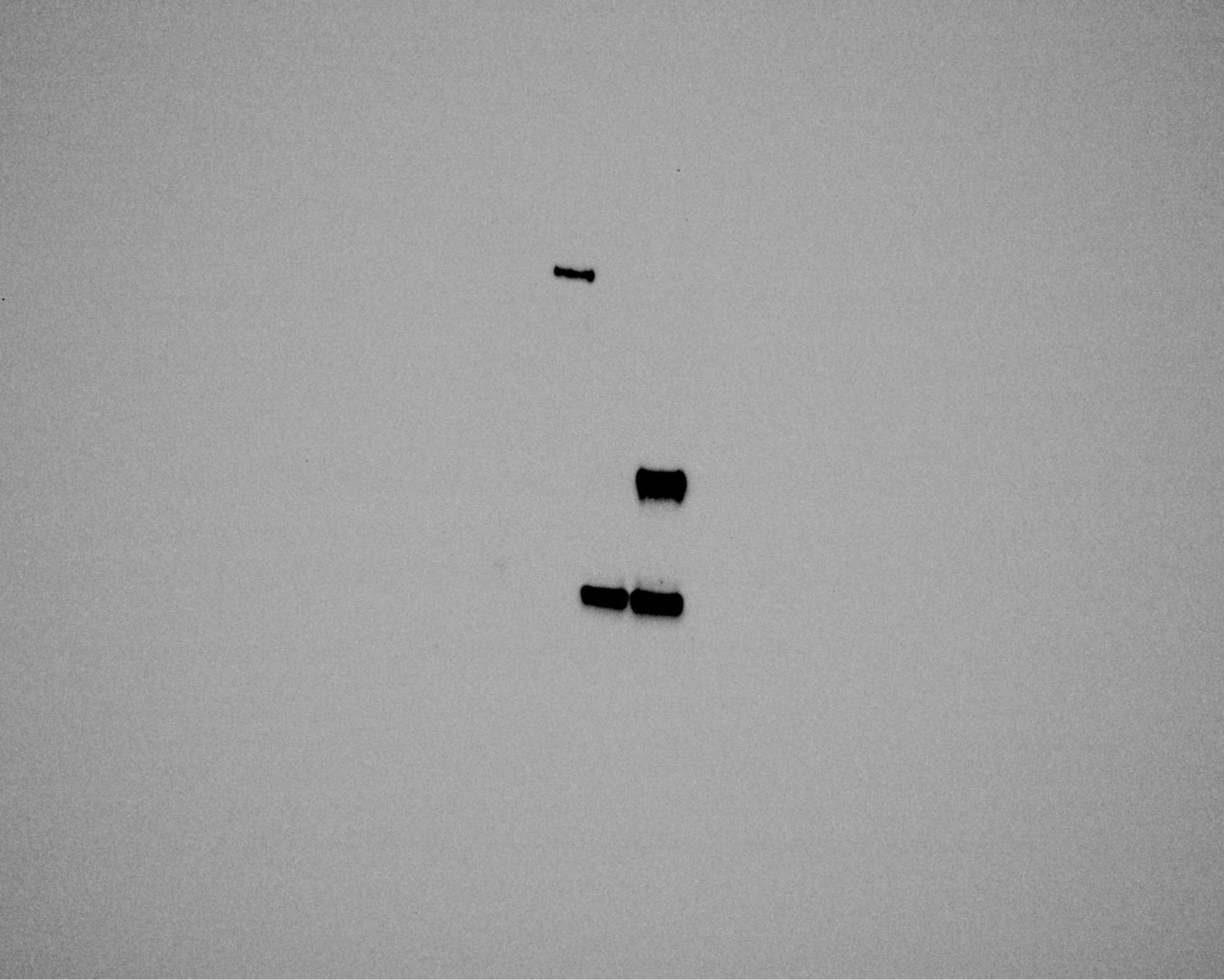

Supplement: Figure 4—figure supplement 1—source data 1. [file elife-77072-fig4-figsupp1-data1.zip › F1S1SD1b.jpg]

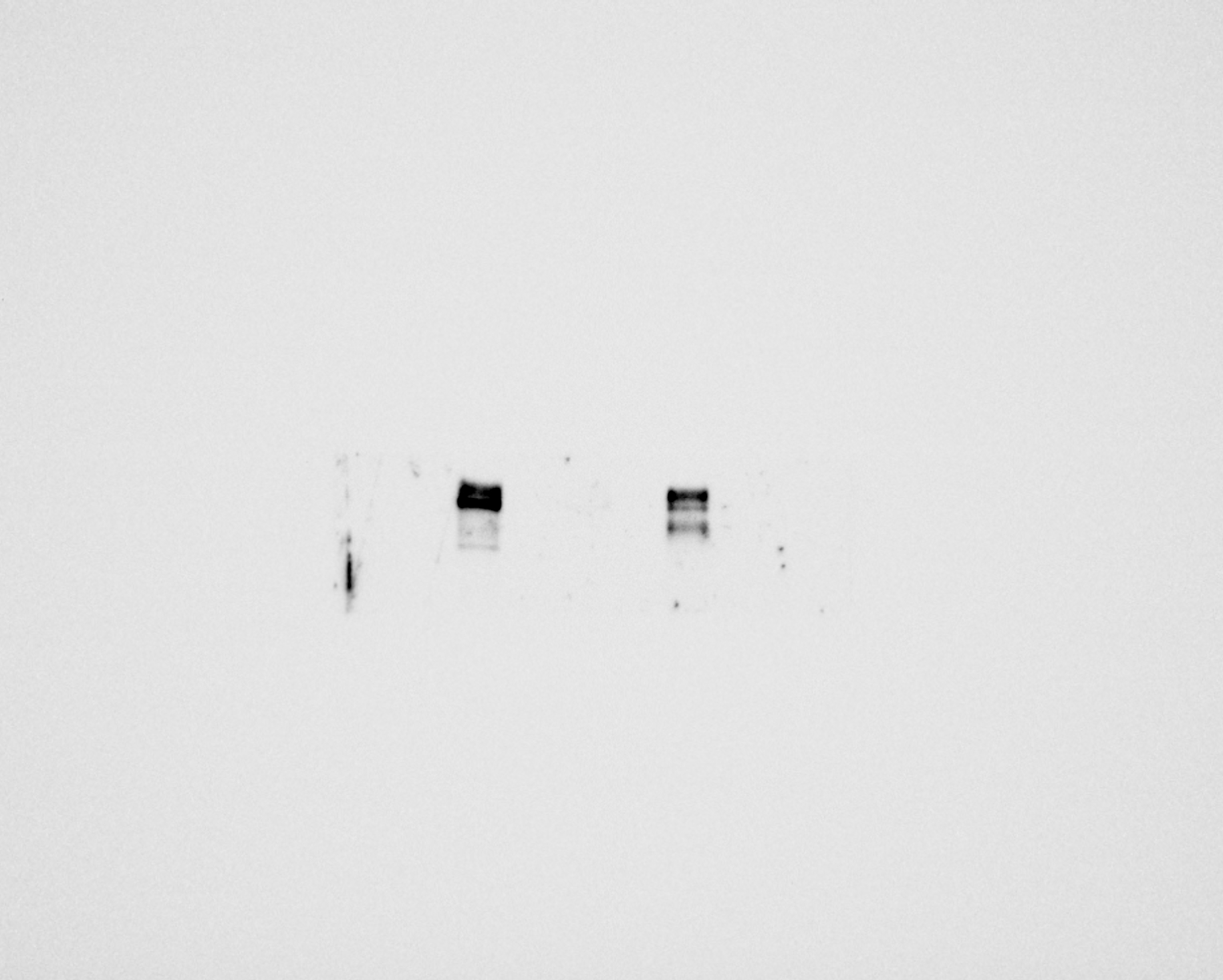

Supplement: Figure 4—figure supplement 1—source data 2. [file elife-77072-fig4-figsupp1-data2.zip › 1.jpg]

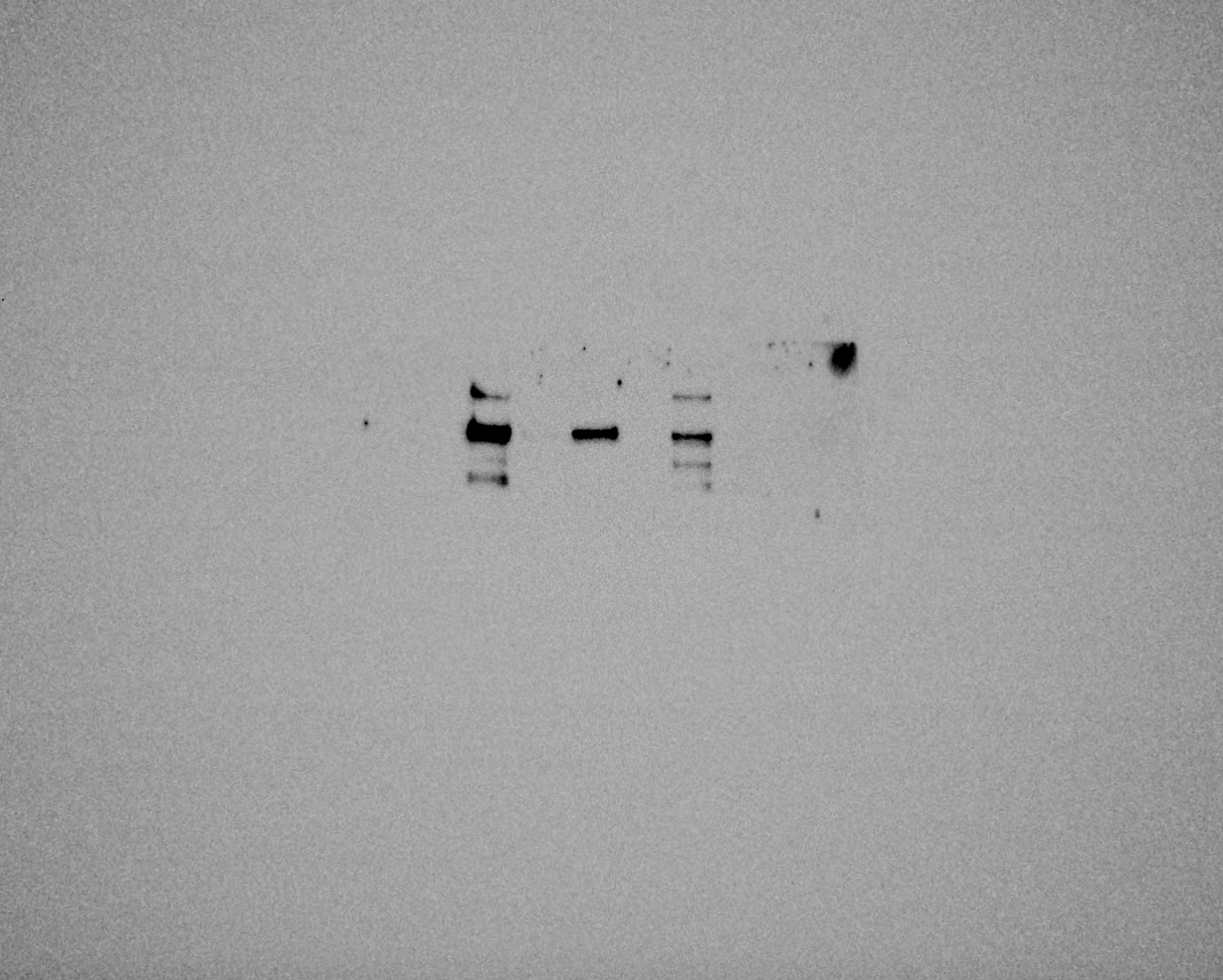

Supplement: Figure 4—figure supplement 1—source data 2. [file elife-77072-fig4-figsupp1-data2.zip › 2.jpg]
