# Supplementary material for: The CIC-ERF co-deletion underlies fusion-independent activation of ETS family member, ETV1, to drive prostate cancer progression
Source: eLife. 2022 Nov 16;11:e77072. doi: 10.7554/eLife.77072 (PMC9668335; doi:10.7554/eLife.77072)
Supplement: Figure 3—figure supplement 1—source data 3. [file elife-77072-fig3-figsupp1-data3.zip › Figure 3 - figure supplement 1 - source data 3/Supplementary figure 3-figure supplement I-Source data.pdf]

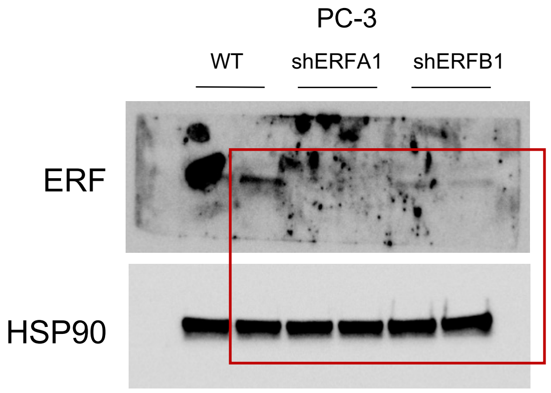

Supplementary figure 3-figure supplement I-Source data: Full length western blot images of ERF and HSP90 in PC3 cells with its variants. Cropped images and description shown in supplementary Figure 2i.
