# Supplementary material for: The CIC-ERF co-deletion underlies fusion-independent activation of ETS family member, ETV1, to drive prostate cancer progression
Source: eLife. 2022 Nov 16;11:e77072. doi: 10.7554/eLife.77072 (PMC9668335; doi:10.7554/eLife.77072)
Supplement: Figure 4—source data 1. — Cropped images and description shown in Figure 4E. [file elife-77072-fig4-data1.zip › Figure 4 - source data 1/figure 4E-source data.pdf]

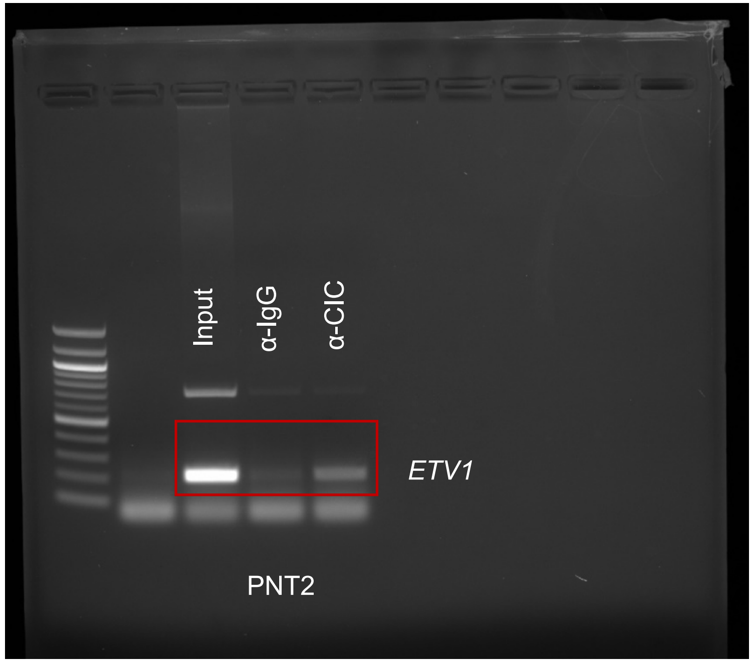


**Figure 4- figure supplement E- source data :** Full length PCR gel images of ETV1 after CIC pull down in PNT2 cells. Cropped images and description shown in Figure 4e.
