# Supplementary material for: The CIC-ERF co-deletion underlies fusion-independent activation of ETS family member, ETV1, to drive prostate cancer progression
Source: eLife. 2022 Nov 16;11:e77072. doi: 10.7554/eLife.77072 (PMC9668335; doi:10.7554/eLife.77072)
Supplement: Supplementary file 2. [file elife-77072-supp2.docx]

**Supplementary Methods**

**ChIP-PCR primers**

The promoter primer sequences used for ChIP-PCR are listed as follows:

**ChIP-Seq datasets**

The following samples were used for VCaP ERF ChIP-Seq data from GEO database: GSE83653.
